# Comparative Impact of Hydroxychloride and Organic Sources of Manganese, Zinc, and Copper in Rearing Diets on Pullet Growth, Tibia Traits, Egg Production, and Eggshell Quality in Lohmann Brown Birds up to 50 Weeks of Age

**DOI:** 10.3390/vetsci11060245

**Published:** 2024-05-29

**Authors:** Reza Akbari Moghaddam Kakhki, Clara Alfonso-Carrillo, Ana Isabel Garcia-Ruiz

**Affiliations:** Poultry Research Centre, Trouw Nutrition R&D, El Viso de San Juan, 45950 Toledo, Spain

**Keywords:** organic mineral source, hydroxychloride source, eggshell quality, long-life hens, young animal feeding

## Abstract

**Simple Summary:**

In this study, the efficacy of different sources of essential minerals, such as zinc, manganese, and copper, in the diets of Lohmann brown pullets was compared. The birds were divided into two groups, with one group receiving diets containing organic sources and the other group receiving diets containing hydroxychloride sources during their early growth phase. No significant differences were observed in growth or bone quality during this phase. However, during the laying period, lower feed intake and egg production were observed in the group fed hydroxychloride sources compared to the organic group. Overall, similar effects on growth and bone quality were seen with both types of mineral sources, but organic sources appeared to improve feed intake and egg production during the laying cycle. This suggests that while hydroxychloride sources hold promise for early growth, organic sources may offer better overall egg production efficiency.

**Abstract:**

(1) Background: This study assessed the efficacy of hydroxychloride sources of zinc (Zn), manganese (Mn), and copper (Cu) compared with organic sources in the rearing diets of Lohmann brown pullets, focusing on pullet performance, tibia quality, egg production, and eggshell quality. (2) Methods: A total of 120 birds (six replications and 10 birds each) received diets with Mn, Zn, and Cu from organic or hydroxychloride sources during the rearing phase. After the onset of lay, birds were fed diets containing oxide/sulfate sources up to 50 weeks of age. (3) Results: no significant differences were observed in growth performance and tibia quality during the rearing phase (*p* > 0.05). From 18 to 24 weeks of age, no carryover effect on egg production performance was observed. However, from 25–50 weeks, pullets fed hydroxychloride sources showed lower feed intake and egg mass compared to the organic group (*p* < 0.05), whereas egg production and eggshell quality remained similar between groups (*p* > 0.05). (4) Conclusions: These findings suggest the potential of hydroxychloride sources in rearing diets without compromising overall growth in the pullet phase and feed efficiency in the laying cycle.

## 1. Introduction

The increasing demand for eggs has subjected individual laying hens to elevated stress, leading to bone deterioration throughout the laying cycle and an increased prevalence of osteoporosis [1]. It is crucial to achieve and sustain peak bone mineral density in structural bones before the onset of lay to mitigate osteoporosis in laying hens [2,3]. Beyond averting osteoporosis, a robust skeletal system significantly influences eggshell quality, as approximately 40% of calcium is supplied by the skeletal system [4]. Eggshell quality is a vital concern in the egg industry, with about 10-15% of eggs produced in poultry farms being lost due to breakage, resulting in considerable economic losses.

The role of micronutrients in poultry nutrition is crucial for ensuring optimal growth, feed efficiency, and the overall health of birds. Zinc (Zn), manganese (Mn), and copper (Cu) are essential trace minerals that play significant roles in various metabolic processes, including enzyme function, antioxidant defense, and maintenance of body functions [5]. For example, Zn is integral to bone and cartilage formation, participating in collagen synthesis and hydroxyapatite crystallization [6]. Copper is vital for bone tensile strength through its role in linking elastin and collagen, with a concurrent need for sufficient copper concentrations to prevent collagen fibril weakening [7]. Manganese is essential for mucopolysaccharides in bone cartilage, and its insufficiency may lead to tibia epiphyseal plate malformation [8].

Traditionally, poultry diets have been supplemented with inorganic salts like sulfate and oxide for trace minerals. However, in recent years, nutritionists have introduced and endorsed organic forms of trace minerals, such as metal amino acid chelates. These organic sources offer advantages over inorganic ones as they are shielded from chemical interactions with other substances in the intestinal environment. This is due to their ability to remain associated with their respective ligands in the acidic gastrointestinal pH, preventing dissociation [9,10].

Hydroxychloride trace minerals are a new class of inorganic minerals, formed by covalent bonds between the elements atoms, multiple hydroxy groups, and chloride ions. This results in a stronger chemical bond compared to the ionic forms, making hydroxychloride trace minerals more soluble at lower pH levels and thus having higher absorption in poultry [11]. Furthermore, hydroxychloride trace minerals have been reported not to bind with dietary constituents, reducing the formation of indigestible complexes [12].

The rearing phase holds particular importance for the optimal development of various body organs, especially impacting bone development, with potential continuing effects on bone health and eggshell quality during the laying phase [2]. Despite significant research conducted on the effects of organic trace minerals, their doses, and sources in laying hens, there remains a notable knowledge gap regarding the efficacy of hydroxychloride sources of Zn, Mn, and Cu in terms of pullet growth, bone development, and subsequently, egg production and eggshell quality during the laying phase. This study aimed to address this gap by evaluating and comparing the effects of a hydroxychloride blend of Zn, Mn, and Cu versus organic sources during the rearing phase on pullet performance and tibia quality, as well as on production performance and eggshell quality during the laying phase.

## 2. Materials and Methods

This study was approved by the Animal Welfare Committee of Trouw Nutrition R&D Poultry Research (internal animal welfare project 13-2021). Birds were handled according to the principles for the care of birds in experimentation [13]. A total of 120 Lohmann Brown Classic day-old pullets were procured from a commercial hatchery and housed in group cages at the experimental pullet facility of the Trouw Nutrition Poultry Research Centre (El Viso De San Juan, Toledo, Spain).

Birds were assigned to one of the two dietary treatments during the pullet phase (up to 16 week) with 6 replications and 10 birds each. All diets were formulated to meet or exceed nutrient requirements based on CVB (2018) [14] and manufactured as a basal mixture per phase from raw materials of the same batch (Table 1). The targeted supplementary Mn, Zn, and Cu levels were 65, 50, and 5 mg/kg, respectively [15]. The experimental diets were offered in crumble form and supplemented with trace mineral premix devoid of Mn, Zn, and Cu. The supply sources of the organic and hydroxychloride minerals were Optimin and Intellibond, respectively (Trouw Nutrition, Tilburg, The Netherlands).

At 16 weeks, eighteen birds from every treatment were moved to a laying facility with individual cages. All birds were fed common laying-hen diets supplemented with Mn oxide, Zn oxide, and Cu sulfate in a mash form to evaluate the carryover effect from the rearing-phase treatments to the laying phase until 50 weeks of age. The diet from the onset of lay to 25 week contained 2750 Kcal/kg AME, 3.3 g/kg dP, and an analyzed value of 166.8 g/kg CP, 40.2 g/kg Ca, and 6.9 g/kg P. The diet from 25 to 50 weeks contained 2800 Kcal/kg AME, 3.1 g/kg dP, and an analyzed value of 155.6 g/kg CP, 45.5 g/kg Ca, and 6.7 g/kg P.

Room temperature was set at 35 °C on arrival and gradually decreased to 20 °C on day 35 until the end of the experimental period. Pullets were kept on a 24 h/d light program (40 lux) for the first week of life, followed by a gradual reduction to 9 h/d light (6 lux) by the 7th week up to 16th week, and followed by an increase to 14 h/d for the rest of the laying period.

Dietary samples were analyzed (Table 1) for dry matter (930.15 (AOAC, 2019)), crude protein (968.06 [16]), ash (942.05 [16]), crude fiber (962.09 [16]), and ether extract (crude fat, 920.39 [16]). The Ca and P levels were determined using the spectrophotometric method with a segmented flow autoanalyzer at MasterLab Trouw Nutrition (Madrid, Spain). The Ca analysis followed the method proposed by Gitelman (1967) based on the reaction of o-cresolphthalein complexone with Ca [17]. The P analysis followed the method described by Murphy and Riley (1962) based on the reaction of phosphate ion with molybdate [18]. The dietary level of Mn, Zn, and Cu were analyzed based on the standard 17,053 of the Spanish Association for Standardization and Certification (2018), using inductively coupled plasma mass spectrometry [19].

The average daily feed intake (ADFI) and body weight (BW) were recorded at the end of each feeding phase during the pullet and laying phases. Hen-day egg production (HDEP) and egg weight (EW) were recorded daily. The feed conversion ratio (FCR) was calculated as the ratio between consumed feed and BW for the rearing period (0 to 16 weeks), and the ratio between ADFI and egg mass (EM; HDEP × weight of salable eggs) for the laying period (18 to 50 weeks). Six birds per treatments were euthanized via cervical dislocation for tibia sampling at 2, 10, and 16 weeks (within the ±95% average BW in that unit).

Right tibia samples were dried at 103 °C for 18h to measure tibia dry weight, followed by ashing in an oven at 550 °C for 12h [20]. The left tibia was used for measuring absolute water, organic matter, carbonate, and phosphate content via thermogravimetry (Universidad de Granada, Granada, Spain) [21]. The percentage of organic matter, carbonate, and phosphate content of the cortical part of the tibia was expressed based on dry matter.

Starting from 25th week, every four weeks, four intact eggs per cage were randomly taken to evaluate egg component yield and eggshell quality (breaking strength, shell weight, and shell thickness). The eggs were then cracked open, and the eggshells were dried using napkins. The eggshell percentage was measured by calculating their ratio to EW. Eggshell breaking strength and thickness were measured using a texture analyzer (TA.XT plus100C, Stable Micro Systems, Godalming, UK) [20]. Shell weight per unit surface area (SWUSA) was calculated by dividing the weight of the eggshell (mg) by the surface area of the eggshell [22]. The albumen weight was calculated via subtraction of the yolk and shell weights from the EW.

Raw data were tested for normality, and outliers (mean ± 3.0 SD) were determined using the INFLUENCE statement of the MIXED procedure of SAS^®^ 9.4 (Cary, NC, USA). During the rearing phase, a cage was considered an experimental unit. During the laying phase, each bird was considered an experimental unit, and production performance and eggshell quality were analyzed as a repeated measurement. The performance and eggshell quality data collected during the laying phase were divided into two distinct phases based on the laying phase diets, including pre-peak (18–24 week) and peak (25–50 week). The percentage of shell-less eggs and broken eggs were analyzed using the MIXED procedure with a binomial distribution. For the rest of the data, the main effect of the treatment was determined using the MIXED procedure of SAS 9.4 (Cary, NC, USA). The differences between the least square means were determined using the Dunnett test. Significance was declared at *p* ≤ 0.05, and tendencies were noted when 0.05 < *p* ≤ 0.10.

## 3. Results

### 3.1. Rearing Phase

There was no difference in ADFI, BW, and FCR between hydroxychloride and organic Mn, Zn, and Cu sources during the rearing phase (*p* > 0.05; Table 2). Mortality was not affected by Mn, Zn, and Cu sources during the rearing phase, with an average of 2.3% during the entire rearing phase.

Additionally, the Mn, Zn, and Cu sources did not affect tibia quality parameters, including length, dry weight, ash content, ash percentage, breaking strength, as well as the percentage of organic matter, carbonate, and phosphate in the cortical part at 2, 10, and 16 weeks of age (*p* > 0.05; Table 3).

### 3.2. Laying Phase

During the pre-peak period (18–24 weeks), there was no carryover effect of different sources of Mn, Zn, and Cu in rearing diets on ADFI, HDEP, EW, EM, FCR, or BW (*p* > 0.05; Table 4). During the peak period (25–50 weeks), the ADFI and EM of hens fed with rearing diets containing hydroxychloride sources of Mn, Zn, and Cu were significantly lower than the organic group (*p* < 0.05; Table 4). However, HDEP, EW, FCR, or BW did not exhibit differences among the treatments (*p* > 0.05). Mortality was not affected by Mn, Zn, and Cu sources during the rearing phase, with an average of 3.6% during the laying phase.

The frequency of occurrence of shell-less eggs and broken eggs was not affected by the sources of Mn, Zn, and Cu in the rearing diet (*p* > 0.05; Table 5). Additionally, the sources of Mn, Zn, and Cu in rearing diets had no effect on the percentage of yolk, albumen, and eggshell quality parameters such as breaking strength, eggshell thickness, and SWUSA at 25 to 50 weeks of age (*p* > 0.05; Table 5).

## 4. Discussion

It is important to point out the discrepancies between the calculated and analyzed content of Mn, Zn, and Cu in the diets. On average, in the organic group, the recovery of Mn, Zn, and Cu was 90%, 81%, and 47% of the calculated values, respectively. In contrast, these values in the hydroxychloride group were 83%, 73%, and 45% of the calculated values for Mn, Zn, and Cu, respectively. The ingredients used in formulating diets can exhibit inherent variability in their mineral content [23]. Natural sources of minerals, such as plant-based ingredients, often show fluctuations due to factors like soil composition, agricultural practices, and processing methods [23]. Additionally, the presence of other dietary components can influence the detectable levels of certain minerals. For example, phytates, present in many plant-based foods, can chelate minerals like iron and zinc [24]. These factors combined can lead to discrepancies between the expected (calculated based on ingredient composition) and actual (analyzed) mineral content in the final diet.

The impact of various Mn, Zn, and Cu sources on growth performance and tibia quality in pullets, especially in a direct comparison between hydroxychloride sources and organic alternatives, has not been previously reported. In line with our findings, Olukosi et al. (2018) reported no significant differences in BW and ADFI in broilers when substituting sulfate sources of Zn (80 mg/kg) and Cu (15 mg/kg) with hydroxychloride sources [25]. Furthermore, they did not observe any change in tibia ash percentage in 35-day-old broilers when substituting sulfate Zn (80 mg/kg) and Cu (15 mg/kg) with hydroxychloride sources. However, it is crucial to note that this study did not directly compare hydroxychloride sources with organic alternatives, focusing primarily on the substitution of inorganic sources with hydroxychloride in broilers [25].

The lack of observed differences when comparing sources may suggest that one potential explanation lies in the inclusion levels of these elements. These levels were suggested to be adjusted at 100, 60, and 5 mg/kg based on the breeder recommendation [26].

Even though the levels of Mn and Zn in the current study were lower than recommended by the breeder, it raises the possibility that the levels utilized in these studies, potentially higher than the actual requirements, could have masked any discernible effects. This emphasizes the importance of investigating the true requirements of these elements in growing birds, as higher inclusion rates may have diminished the potential to observe significant impacts on growth and tibia quality.

The substitution of organic sources of Mn, Zn, and Cu with hydroxychloride sources in rearing diets did not affect the growth performance of pullets and their bone attributes; however, it showed a long-lasting effect on egg production performance criteria during the laying cycle. It is noteworthy that the birds in this trial achieved their peak egg production six weeks earlier than the age reported by the breeder [26]. This may be attributed to the fact that the pullets had a higher body weight (1659 g) as a result of being fed crumble diets, which exceeded the recommended range of 1290–1370 g, as prescribed by the breeder. Previous studies have suggested that higher BW in pullets is associated with larger ovaries, leading to an earlier onset of lay and higher early egg production [27,28].

The carryover effect of trace mineral sources in rearing diets on egg production performance has not been previously reported. Additionally, the impact of trace mineral sources in laying diets on egg production criteria has yielded inconsistent results. For example, Olukosi et al. (2019) found no significant effect on parameters such as HDEP, FCR, EM, eggshell thickness, and eggshell percentage. However, they observed a reduction in EW and the percentage of cracked eggs when substituting the sulfate source of Mn, Zn, and Cu with hydroxychloride sources in 24 week Lohmann brown hens [29].

Conversely, Jiang et al. (2021) did not observe a significant difference in HDEP, EW, FCR, and broken egg percentage when substituting the sulfate source of Mn, Zn, and Cu with hydroxychloride sources [12]. In a longer-term trial spanning from 20 to 70 weeks, the substitution of organic sources for inorganic Zn (30 mg/kg) and Mn sources (50 mg/kg) did not lead to any significant effect on egg production criteria [30].

In the current study, there was no difference in ADFI during the pullet phase using hydroxychloride and organic sources, but differences were observed in ADFI during the laying phase. This suggests that the change in ADFI occurred due to switching from hydroxychloride and organic forms of Zn, Mn, and Cu in the rearing diets to oxide/sulfate sources in the laying diet. Higher ADFI in hens fed organic sources of Mn, Zn, Cu in the rearing phase can be explained by their higher EM compared to those fed with hydroxychloride sources in the rearing phase. However, FCR was not different between the treatments, showing that using hydroxychloride sources of these elements in rearing diets was as effective as organic sources in supporting feed efficiency in the laying phase up to 50 weeks.

Currently, there is limited information on the roles of Mn and Cu in regulating feed intake in poultry. However, it has been demonstrated that Zn deficiency in broilers can decrease their ADFI [9]. Studies on pigs have shown that dietary Zn can enhance feed intake by stimulating the orexigenic signaling action of ghrelin, a hormone present in the stomach and plasma. In its acylated form, ghrelin can cross the blood–brain barrier and stimulate appetite by acting on orexigenic neurons [31].

The study by Pereira et al. (2020) examined the effects of mixing 30 mg/kg of Zn, 30 mg/kg of Mn, and 5.75 mg/kg of Cu from organic sources with 40 mg/kg of Zn, 40 mg/kg of Mn, and 2.25 mg/kg of Cu from inorganic sources in the rearing and laying diets of Lohmann brown hens for up to 26 weeks [32]. Although neither EM nor BW gain were affected by the dietary treatments, ADFI was found to be higher in laying hens fed with a mixture of organic and inorganic sources than those fed with only inorganic sources. In addition, Bakhshalinejad et al. (2024) compared the efficacy of a blend of 80% hydroxychloride and 20% organic sources of Mn, Zn, and Cu versus 100% organic sources in broiler breeders during weeks 42 to 63 [33]. Their results showed that using the blend of hydroxychloride and organic sources led to lower ADFI and higher HDEP, EM, and EW compared to using only organic sources [33].

Substitution of inorganic sources of Mn, Zn, and Cu with hydroxychloride sources in laying hens has been reported to improve eggshell quality [12]. Additionally, an improvement in eggshell quality in broiler breeders was observed in response to using a blend of 80% hydroxychloride and 20% organic sources of Mn, Zn, and Cu instead of 100% organic sources [33].

The potential for Mn, Zn, and Cu to improve eggshell quality is associated with their involvement in critical enzymes for eggshell formation. Manganese acts as an activator of enzymes involved in the synthesis of glycosaminoglycans and glycoproteins, which contribute to the formation of the eggshell’s organic matrix [34,35]. Zinc is a cofactor of carbonic anhydrase inhibitors involved in eggshell formation, while copper is an integral part of the lysyl oxidase enzyme important for collagen formation in the eggshell membrane [36]. The lack of effect on eggshell quality in the current study can be explained by switching hydroxychloride or organic sources in rearing diets to sulfate/oxide sources in laying diets, as well as the lack of effect of Mn, Zn, and Cu sources on the skeletal system during the pullet phase.

A significant gap exists in our understanding of the relative efficacy of hydroxychloride trace element sources compared to organic sources in pullet nutrition. It is noteworthy that our investigation into this comparative aspect is particularly relevant given the potential cost implications. Usually, hydroxychloride sources tend to be less expensive compared to organic sources. If hydroxychloride sources demonstrate comparable efficacy to organic alternatives, the former, being more cost-effective, could present a promising avenue for producers to enhance profit margins. Using organic sources of Mn, Zn, and Cu in rearing diets and transitioning to sulfate/oxide sources in laying diets led to higher ADFI and EM compared to their hydroxychloride counterparts. However, similar FCR between the two groups demonstrates the similar potential of hydroxychloride and organic sources in supporting efficient FCR in the laying cycle.

## 5. Conclusions

This study found that replacing organic with hydroxychloride sources of Mn, Zn, and Cu in the rearing diets did not impact the growth performance and tibia quality in Lohmann Brown pullets. During the transition to the laying cycle and the switch to Mn oxide, Zn oxide, and Cu sulfate in both treatments, hens fed organic sources during their rearing phase exhibited higher ADFI and EM compared to the hydroxychloride group. This suggests the potential long-lasting effects of Mn, Zn, and Cu sources from the rearing diets throughout the laying cycle. Nevertheless, it is important to take into account the potential impact of increased BW resulting from use of crumble feed, as this could diminish the detectability of variations. Further studies are warranted to understand the long-term effects of using various levels of trace minerals from different sources throughout the laying cycle, as well as to assess the efficiency of these birds in retaining these elements.

## Figures and Tables

**Table 1:** Experimental ingredient and chemical diets composition with different manganese, zinc, and copper sources during the rearing phase.

Items	Starter (1–3 Week)		Grower (4–10 Week)		Developer (11–16 Week)	
	Organic	Hydroxychloride	Organic	Hydroxychloride	Organic	Hydroxychloride
Ingredient, g/kg						
Corn ^1^	400.00	400.00	400.00	400.00	611.74	611.74
Wheat	225.55	225.55	108.34	108.34	60.00	60.00
Soybean meal, CP = 480 g/kg	285.59	285.59	253.23	253.23	54.56	54.56
Sunflower meal, CP = 310 g/kg	-	-	-	-	120.00	120.00
Barley	-	-	150.00	150.00	-	-
Barely Straw	-	-	-	-	40.95	40.95
Potato Protein	-	-	-	-	43.38	43.38
Wheat Bran Fine	-	-	20.29	20.29	-	-
Soybean oil	17.38	17.38	5.00	5.00	5.00	5.00
Oats hulls	30.00	30.00	30.00	30.00	30.00	30.00
Calcium carbonate (fine)	16.77	16.22	13.18	12.64	8.58	8.10
Monocalcium phosphate	4.81	4.81	2.25	2.25	2.12	2.12
Sepiolites Elite SPLF	4.39	4.94	6.89	7.43	13.79	14.27
Salt (NaCl)	2.78	2.78	3.03	3.03	2.51	2.51
Na Bicarbonate	2.30	2.0	0.47	0.47	0.85	0.85
Dl-Methionine 99%	2.23	2.23	1.22	1.22	0.16	0.16
L-Lysine HCl 98%	1.39	1.39	0.10	0.10	0.37	0.37
AXTRA XAP ^2^	1.00	1.00	1.00	1.00	1.00	1.00
L-Threonine 98%	0.82	0.82	-	-	-	-
Premix (organic) ^3,4^	5.00	-	5.00	-	5.00	-
Premix (hydroxychloride) ^3,4^		5.00	-	5.00	-	5.00
Calculated nutrients (analyzed nutrients ^5^)	-	-	-	-	-	-
AME, Kcal/kg	2850	2850	2750	2750	2700	2700
Crude protein, g/kg	190.45 (198.1)	190.45 (194.8)	177.54 (178.8)	177.54 (177.5)	150.00 (149.3)	150.00 (148.3)
Crude fiber, g/kg	34.18 (32.6)	34.18 (31.0)	38.37 (36.5)	38.37 (39.0)	70.93 (68.8)	70.93 (67.4)
Crude fat, g/kg	43.98 (34.5)	43.98 (35.0)	32.34 (23.5	32.34 (22.0)	35.46 (25.5)	35.46 (23.5)
Dig Lysine, g/kg ^6^	9.50	9.50	7.80	7.80	6.20	6.20
Dig methionine, g/kg	4.98	4.98	3.57	3.57	2.80	2.80
Dig methionine+ cystine, g/kg	7.70	7.70	6.01	6.01	4.84	4.84
Dig threonine, g/kg	6.84	6.84	5.30	5.30	4.20	4.20
Calcium, g/kg	9.00 (7.1)	9.00 (7.7)	7.60 (5.8)	7.60 (5.5)	6.70 (5.8)	6.70 (5.5)
Digestible phosphorus, g/kg ^7^	3.80	3.80	3.30	3.30	2.90	2.90
Total phosphorus, g/kg	4.53 (4.4)	4.53 (4.1)	3.99 (4.0)	3.99 (3.9)	3.44 (3.1)	3.44 (2.9)
Sodium, g/kg	1.80	1.80	1.40	1.40	1.40	1.40
Chloride, g/kg	2.50	2.50	2.50	2.50	2.50	2.50
Potassium, g/kg	8.77	8.77	8.58	8.58	6.42	6.42
Magnesium, g/kg	1.61	1.61	1.61	1.61	1.63	1.63
Manganese, mg/kg	110 (101)	110 (88)	111 (103)	111 (96)	105 (90)	105 (87)
Zinc, mg/kg	108 (86)	108 (78)	109 (93)	109 (83)	111 (86)	111 (80)
Copper, mg/kg	20 (10)	20 (9)	21 (9)	21 (9)	21 (10)	21 (10)

^1^ A total of 30 g/kg was supplied in a coarse form at 6 mm. ^2^ Xylanase 3.2.1.8 and glucanase 3.2.1.6 (Dupont, Wilmington, Delaware, United States). ^3^ There were two types of experimental premixes, namely, organic and hydroxychloride, which differed only in the source of manganese, zinc, and copper. Supplementary organic sources: Optimin Mn, Zn, and Cu (Trouw Nutrition, Tilburg, The Netherlands); for hydroxychloride: Intellibond (Trouw Nutrition, Tilburg, The Netherlands). ^4^ Supplied 1000 FTU phytase, 10,000 IU/kg of vitamin A, 3000 IU/kg of vitamin D3, 30 IU/kg of vitamin E, 3 mg/kg of vitamin K3, 2 mg/kg of vitamin B1, 8 mg/kg of vitamin B2, 8 mg/kg of vitamin B2, 40 mg/kg of B3, 14 mg/kg of B5, 4 mg/kg of B6, 100 mcg/kg of B7, 1 mg/kg of B9, 25 mcg/kg of B12, 300 mg/kg of choline, 100 mg/kg of betaine, 65 mg/kg of manganese, 50 mg/kg of zinc, 25 mg/kg of iron, 5 mg/kg of copper, 1.9 mg/kg of iodine, and 0.15 mg/kg of selenium to the final diet. ^5^ The average of the samples and duplicated analysis. ^6^ Apparent fecal digestibility for all the amino acid values. ^7^ CVB Feed Table 2019.

**Table 2:** The effect of different sources of manganese, zinc, and copper on growth performance during the rearing phase ^1^.

Items	Organic	Hydroxychloride	SEM (*n* = 6) ^2^	*p*-Value
Starter (1 to 3 weeks)				
Body weight, g	198	199	3.0	0.857
Daily feed intake, g/bird/day	13.6	14.0	0.26	0.381
Feed conversion ratio	1.890	1.933	0.0262	0.153
Grower (4 to 10 weeks)				
Body weight, g	1104	1105	12.7	0.999
Daily feed intake, g/bird/day	54.5	55.0	0.80	0.629
Feed conversion ratio	2.948	2.971	0.0271	0.555
Developer (11 to 16 weeks)				
Body weight, g	1659	1659	11.3	0.972
Daily feed intake, g/bird/day	82.8	84.6	2.01	0.551
Feed conversion ratio	6.291	6.424	0.1869	0.450

^1^ Manganese, zinc, and copper. Supplementary organic sources: Optimin Mn, Zn, and Cu (Trouw Nutrition, Tilburg, The Netherlands); for hydroxychloride: Intellibond (Trouw Nutrition, Tilburg, The Netherlands). The targeted supplementary Mn, Zn, and Cu levels were 65, 50, and 5 mg/kg of diet. ^2^ Data are least squares means of six replications and 10 birds each.

**Table 3:** The effect of different sources of manganese, zinc, and copper on tibia quality at different ages ^1^.

Items	Organic	Hydroxychloride	SEM (*n* = 6) ^2^	*p*-Value
2 weeks				
Length, mm	44	43	0.4	0.127
Dry weight, g	0.65	0.56	0.145	0.155
Ash content, g	0.29	0.24	0.020	0.123
Ash, %	44.17	43.82	0.494	0.643
Breaking strength, g	2954	2817	199.2	0.501
Cortical composition, % of DM				
Organic matter	42.1	42.1	2.52	0.999
Carbonate	2.2	3.3	1.50	0.482
Phosphate	55.7	54.6	2.10	0.823
10 weeks				
Length, mm	99	99	1.1	0.749
Dry weight, g	6.28	6.14	0.185	0.625
Ash content, g	2.18	2.33	0.134	0.510
Ash, %	37.90	37.94	0.435	0.957
Breaking strength, g	15,995	16,122	1120	0.911
Cortical composition, % of DM				
Organic matter	35.8	35.7	1.53	0.970
Carbonate	5.4	7.3	4.36	0.520
Phosphate	58.2	56.6	2.95	0.615
16 weeks				
Length, mm	120	121	1.6	0.641
Dry weight, g	9.82	10.25	0.243	0.243
Ash content, g	3.22	3.36	0.056	0.142
Ash, %	32.82	32.82	0.479	0.992
Breaking strength, g	15,298	15,399	654.3	0.879
Cortical composition, % of DM				
Organic matter	29.4	27.6	1.31	0.239
Carbonate	29.6	28.4	2.42	0.655
Phosphate	41.0	43.9	1.56	0.128

^1^ Manganese, zinc, and copper. Supplementary organic sources: Optimin Mn, Zn, and Cu (Trouw Nutrition, Tilburg, The Netherlands); for hydroxychloride: Intellibond (Trouw Nutrition, Tilburg, The Netherlands). The targeted supplementary Mn, Zn, and Cu levels were 65, 50, and 5 mg/kg of diet. ^2^ Data are least squares means of six pullets per treatment for tibia quality (one bird per cage).

**Table 4:** The effect of different sources of manganese, zinc, and copper during the pullet phase on egg production performance in Lohmann Brown birds at 18 to 50 weeks of age ^1^.

Items	Organic	Hydroxychloride	SEM	*p*-Value
18 to 24 weeks			(*n* = 18) ^2^	
Daily feed intake, g	110	109	2.3	0.629
Hen-day egg production, %	95.98	97.86	1.130	0.128
Egg weight, g	56.42	56.69	1.064	0.801
Egg mass, g/d	52.43	53.19	1.541	0.651
Feed conversion ratio	2.408	2.104	0.211	0.200
Body weight at the end of 24 weeks, g	1906	1896	59.1	0.873
25 to 50 weeks			(*n* = 16) ^3^	
Daily feed intake, g	122	118 *	1.7	0.006
Hen-day egg production, %	96.66	96.24	0.610	0.479
Egg weight, g	64.94	63.59	0.856	0.122
Egg mass, g/d	62.46	60.82 *	0.637	0.011
Feed conversion ratio	1.975	1.954	0.0227	0.337
Body weight at the end of 49 weeks, g	2023	1989	93.5	0.724

* Indicates that the value in the hydroxychloride group is significantly different from that of the organic group (*p* < 0.05). There was no effect of treatment × week in any parameters (*p* > 0.05). ^1^ Manganese, zinc and copper. Supplementary organic sources: Optimin Mn, Zn, and Cu (Trouw Nutrition, Tilburg, The Netherlands); for hydroxychloride: Intellibond (Trouw Nutrition, Tilburg, The Netherlands). The targeted supplementary Mn, Zn, and Cu levels were 65, 50, and 5 mg/kg of diet. ^2^ Data are least squares means of 18 replications (18 birds) per treatment from 18 to 24 weeks of age. ^3^ Data are least squares means of 16 replications (16 birds) per treatment from 25 to 50 weeks of age.

**Table 5:** The effect of different sources of manganese, zinc, and copper during the pullet phase on the frequency of shell-less, broken eggs, egg component percentage, and eggshell quality in Lohmann Brown birds at 25 to 50 weeks of age ^1^.

Items	Organic	Hydroxychloride	SEM (*n* = 16) ^3^	*p*-Value
Shell-less egg, % ^2^	0.68	0.71	0.119	0.502
Broken eggs, % ^2^	0.25	0.13	0.308	0.959
Albumin Percentage	64.67	64.74	0.565	0.900
Yolk percentage	25.53	25.24	0.507	0.562
Eggshell percentage	9.97	10.15	0.139	0.192
Breaking strength, g	6451	6464	225.0	0.956
Eggshell thickness, mm	0.375	0.378	0.0081	0.769
Shell weight per unit surface area ^2^	85.57	86.63	1.199	0.381

^1^ Manganese, zinc, and copper. Supplementary organic sources: Optimin Mn, Zn, and Cu (Trouw Nutrition, Tilburg, The Netherlands); for hydroxychloride: Intellibond (Trouw Nutrition, Tilburg, The Netherlands). The targeted supplementary Mn, Zn, and Cu levels were 65, 50, and 5 mg/kg of diet. ^2^ Data were analyzed with the binomial distribution as a number of shell-less or broken eggs relative to the total number of laid eggs. ^3^ Data are least squares means of 16 replications (16 birds) per treatment with 4 eggs per cage collected every four weeks (28 eggs per cage in total).

## Data Availability

The datasets presented in this article are not readily available because the data are part of an ongoing study and due to technical limitations. Requests to access the datasets should be directed to R.A.M.K.
